# Order of Inoculation during *Heligmosomoides bakeri* and *Hymenolepis microstoma* Coinfection Alters Parasite Life History and Host Responses

**DOI:** 10.3390/pathogens2010130

**Published:** 2013-03-01

**Authors:** Paul R. Clark, W. Timothy Ward, Samantha A. Lang, Alaa Saghbini, Deborah M. Kristan

**Affiliations:** 1 Department of Biological Sciences, California State University San Marcos, San Marcos, CA 92096, USA; E-Mails: PaulClark@students.rossu.edu (P.R.C.); WilliamWard@students.rossu.edu (W.T.W.); lang014@cougars.csusm.edu (S.A.L.); AlaaSaghbini@students.rossu.edu (A.S.); 2 Current Address: Ross University, School of Medicine, NJ 08902, USA

**Keywords:** coinfection, Nematode, *Heligmosomoides bakeri*, Cestode, *Hymenolepis microstoma*, life history, host plasticity

## Abstract

Parasite life history may differ during coinfection compared to single infections, and the order of infection may be an important predictor of life history traits. We subjected laboratory mice (*Mus musculus*) to single and coinfections with *Heligmosomoides bakeri* and *Hymenolepis microstoma* and measured life history traits of worms and also hepatobiliary and morphological responses by the host. We found that fewer *H. bakeri* larvae established, and adult worms were shorter and produced fewer eggs during a coinfection where *H. microstoma* occurred first. *H. microstoma* grew more and released more eggs after simultaneous inoculation of both parasites compared to a single *H. microstoma* infection, despite similar worm numbers. Mouse small intestine mass, but not length, varied with coinfection and bile duct mass was largest when *H. microstoma* was given alone or first. Mouse serum alkaline phosphatase levels were greatest for mice infected with *H. microstoma* only but did not vary with number of scolices; no change in mouse serum alanine transaminase levels was observed. Overall, the order of coinfection influenced life history traits of both *H. bakeri* and *H. microstoma*, but changes in survival, growth, and reproduction with order of inoculation were not consistent between the two parasites.

## 1. Introduction

Parasite life history depends on numerous abiotic (e.g., temperature, moisture) and biotic (e.g., food availability, host immune responses) factors which may impact larval establishment and growth as well as subsequent adult survival and reproduction. One important component of a parasite’s biotic environment is the presence of other parasites. Helminth parasite infection of wild animals is widespread [1] and hosts often are infected with multiple helminth species [2]. Such infections with multiple parasite species can have important consequences for host-parasite interactions and for parasite-parasite interactions. 

The dynamics of coinfection are clearly multi-faceted and not surprisingly the documented outcomes of these relationships vary such that infection with one parasite species may enhance (e.g., [3,4,5]), diminish (e.g., [6,7]), or not affect (e.g., [8,9]) the establishment or survival of another parasite species. How parasite species respond to the presence of each other varies tremendously [10] and may be influenced by the particular species involved, location of the parasites in the host, duration of coinfection [11], strain differences within a parasite species [12], or which parasite infects the host first [13,14]. Because of the ability for some helminth parasites to modulate the host immune response (reviewed by [15,16,17]), their presence during coinfection is especially interesting as they may modify infectivity of microparasites [18] or other helminths [19]. We examined coinfection of laboratory mice (*Mus musculus*) by two helminth species, *Heligmosomoides bakeri* (Nematoda) (following nomenclature proposed by [20]) and *Hymenolepis microstoma* (Cestoda). These parasites share the laboratory mouse small intestine and may have direct and indirect effects on each other. 

Host immune responses to an ongoing infection may influence success of subsequent infections. Although there is variation among mouse strains, *H. bakeri* typically elicits an enhanced T-helper cell Type 2 (Th2) response [21,22]; this response may be moderated by increased T-regulatory cell [23] production which is known to mediate T effector cells. In addition, increased antibody production *via* B cells is as an essential component of a protective memory response against *H. bakeri* [24]. *H. microstoma* induces increased antibody production [25,26] and mastocytosis [27,28]; while direct evidence of T cell changes are not described, mice infected with *Hymenolepis nana* show increased numbers of lymphocytes [29] and *H. microstoma* infection may also alter profiles of effector and/or regulatory T cells. While both parasites elicit some overlapping host immune responses, each species also causes unique host immune changes, which means that predicted outcomes of coinfection based on host immune responses are not straightforward. 

An earlier examination revealed that order of coinfection was important for adult *H. bakeri* but not for adult *H. microstoma* [30]. Specifically, there were fewer *H. bakeri* worms when mice were infected with *H. microstoma* first than when mice were infected with *H. microstoma* second or during a *H. bakeri*-only infection. It is not known, however, if the observed difference in *H. bakeri* infection intensity was due to decreased larval establishment or decreased adult survival. Greater *H. microstoma* mass occurred during coinfection, regardless of the order of infection, than during a single *H. microstoma* infection [30]. It was not determined if greater *H. microstoma* mass was due to more worms (scolices were not counted) or greater growth of the same number of worms. Given that the order of coinfection affected *H. bakeri* but not *H. microstoma* [30], the effects of a simultaneous infection on life history traits of these parasites also may reveal important information on parasite interactions during larval establishment and growth, as well as subsequent adult survival and reproduction. 

Our study had three goals. First, we examined if a high or low *H. microstoma* infection intensity would differentially affect *H. bakeri* larval establishment and site selection in the small intestine compared to a *H. bakeri* only infection. Because *H. microstoma* attach in the mouse bile duct with their strobila passing through the common bile duct and into the small intestine, they may disrupt flow of bile into the small intestine [31]. This is important because bile is the chemical cue for *H. bakeri* larvae to embed in the host duodenum [32]. Also, *H. microstoma* induce a mast cell response in the host [27] and mast cells can negatively affect *H. bakeri* [33]. Therefore, we hypothesized that presence of *H. microstoma* would alter survival and site selection of fourth stage larvae (L_4_) *H. bakeri* because the typical concentration of bile in the duodenum would be disrupted. We predicted fewer numbers of *H. bakeri* L_4_ during *H. microstoma* coinfection and that *H. bakeri* L_4_ would be distributed more broadly throughout the small intestine; these effects were expected to be more pronounced for high compared to low infections of *H. microstoma*. Second, we examined if order of infection would affect adult size (as a reflection of larval and early adult growth), survival, and reproduction for both *H. microstoma* and *H. bakeri*. We hypothesized that if *H. bakeri* occurred first that *H. microstoma* would survive better and that, after accounting for the number of *H. microstoma* worms, *H. microstoma* would attain greater mass due to the immunosuppressive ability of *H. bakeri* which would make for a more favorable small intestine environment. We also hypothesized that if *H. microstoma* occurred first that fewer adult *H. bakeri* would occur (due to fewer surviving larvae that established in non-preferred intestinal habitat) and that adult worms would be smaller and females would produce fewer eggs. We predicted intermediate responses when both parasites were given simultaneously. Third, we examined if coinfection would alter host tissue responses to *H. microstoma* as related to bile duct occlusion and potential associated liver damage, as well as changes in small intestine morphology. While increased bile duct mass [34] and liver pathology [35] during *H. microstoma* infection is well-known, it is not clear if presence of *H. microstoma* induces cholestasis *via* extrahepatic biliary obstruction. Biliary obstruction, as evidenced by high levels of serum alkaline phosphatase (ALP) [36], can contribute to liver damage [37] which can be detected by high levels of serum alanine transaminase (ALT) [38]. We hypothesized that ALP and ALT would be greater in mice with single infections or coinfections of *H. microstoma* than in nematode-only infected mice and that these enzyme levels would be positively correlated with the number of *H. microstoma* worms. We also examined if coinfection differentially affected small intestine morphology. Small intestine length and mass increase during single infections of *H. bakeri* [39] and the duodenum becomes thicker [27] and weighs more [31] during single infections of *H. microstoma*. We hypothesized that small intestine mass and length would increase more during coinfection than during single infections, regardless of order of inoculation, because of potential additive effects of both parasites on host small intestine morphology.

## 2. Results and Discussion

A description of the experimental design and definitions of abbreviations for experimental groups are noted in Methods Section 3.1. below. 

### 2.1. Parasite Life History

**Figure 1:**
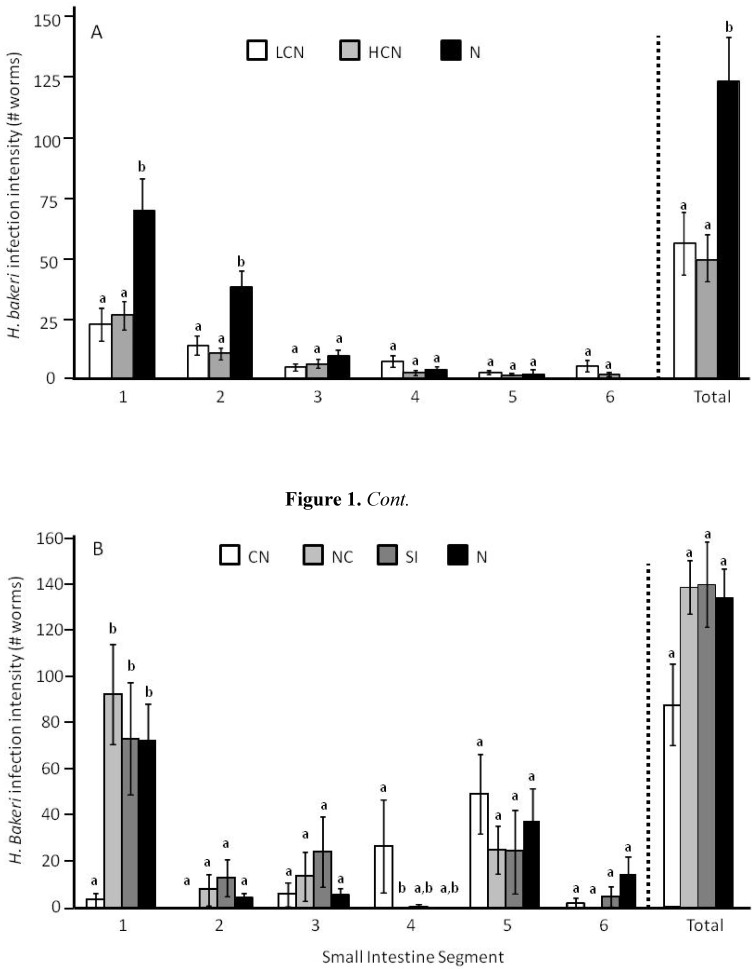
Infection intensity of the nematode *Heligmosomoides bakeri* of fourth larval stage (L_4_) (**A**) and adults (**B**) in each of six equal length small intestine segments (Segment 1 starts immediately distal to the stomach at the pyloric sphincter and Segment 6 ends immediately proximal to the cecum at the ileocecal valve) and the summed total across all segments for different treatment groups (LCN: low cestode infection, then nematode; HCN: high cestode infection, then nematode; N: nematode only; C: cestode only; CN: cestode then nematode; NC: nematode then cestode; SI: simultaneous inoculation). Within a small intestine segment, bars that share the same letter do not differ from each other at *P* < 0.05. Values are means ± 1 standard error.

**Figure 2:**
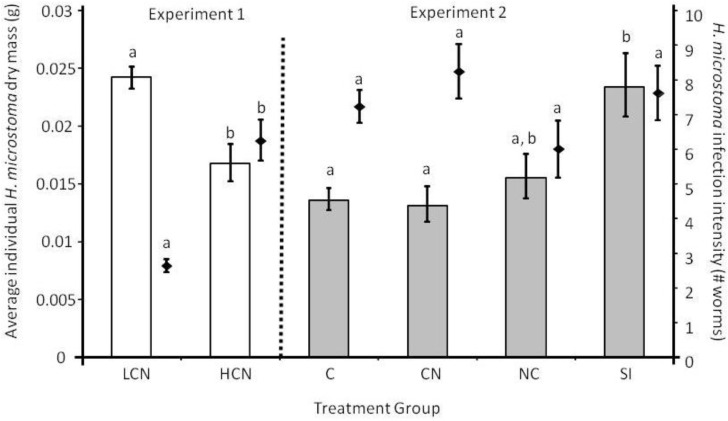
Infection intensity of the cestode *Hymenolepis microstoma* (right y-axis denoted by diamonds) and average individual dry worm mass (left y-axis denoted by bars) for different treatment groups (LCN: low cestode infection, then nematode; HCN: high cestode infection, then nematode; C: cestode only; CN: cestode then nematode; NC: nematode then cestode; SI: simultaneous inoculation). Within an experiment, bars or diamonds that share the same letter do not differ from each other at *P* < 0.05. Values are means (backtransformed from Log_10_ for worm mass in Experiment 2) ± 1 standard error.

Survival of *H. bakeri* L_4_ was less during coinfection (LCN or HCN) than in a *H. bakeri*-only infection regardless of the number of *H. microstoma* (F_2,21_ = 8.54, *P* = 0.002; Figure 1A). In contrast, survival of adult *H. bakeri* worms did not vary among treatment groups (F_3,34_ = 2.5, *P* = 0.073; Figure 1B). However, if only coinfected mice were considered (*i.e.*, CN, NC, SI), then there were marginally fewer *H. bakeri* worms in CN mice compared to NC and SI mice (F_2,20_ = 3.4, *P* = 0.052). As expected, there were 58% fewer *H. microstoma* worms in LCN than in HCN mice (T = 5.87, df = 8.3, *P* < 0.0001; Figure 2) but *H. microstoma* infection intensity did not differ among treatment groups (F_3,41_ = 1.37, *P* = 0.265; Figure 2) when similar numbers of cysticercoids were used during inoculation in Experiment 2.

Site selection along the small intestine for L_4_ that survived varied with treatment group (F_12,34_ = 2.57, *P* = 0.015) because nematode only infected mice (N) had more L_4_ in segments 1 (F_2,21_ = 55.76, *P* < 0.0001) and 2 (F_2,21_ = 63.09, *P* < 0.0001) but fewer in segment 6 (F_2,21_ = 8.23, *P* = 0.009) than coinfected mice which did not differ between each other (Figure 1A). When examined based on percentage of total worms, the distribution of L_4_ still varied among treatment groups (arcsine-square root transformed; F_12,34_ = 3.18, *P* = 0.004) because LCN mice had a greater percentage of L_4_ in Segment 4 (F_2,21_ = 5.39, *P* = 0.013) and Segment 6 (F_2,21_ = 8.46, *P* = 0.002) than mice in other treatment groups, with no differences in percent distribution for the other segments (Segment 1: F_2,21_ = 2.91, *P* = 0.077; Segment 2: F_2,21_ = 1.806, *P* = 0.189; Segment 3: F_2,21_ = 0.99, *P* = 0.389; Segment 5: F_2,21_ = 1.98, *P* = 0.164). The distribution of adult *H. bakeri* along the small intestine varied among treatment groups (reciprocal transformed; F_18, 93_ = 2.20, *P* = 0.008; Figure 1B); this was because for Segment 1 CN mice had fewer *H. bakeri* worms than the other treatment groups (F_3,34_ = 24.63, *P* < 0.0001) and because of more modest differences in number of *H. bakeri* worms among treatment groups for segment 4 (F_3,34_ = 3.17, *P* = 0.037; Figure 1B). When examined based on percentage of total worms, the distribution of adult worms still varied among treatment groups (arcsine-square root transformed; F_18,93_ = 1.82, *P* = 0.033). Using Tahame’s post-hoc test (due to unequal variances among treatment groups for some segments), CN mice had a smaller percentage of total worms in Segment 1 compared to all other treatment groups (*P* < 0.05) which did not differ from each other. Percent distribution among Segments 2–6 did not vary among any treatment groups at *P* < 0.05. 

Sex ratio of L_4_ (F_2,21_ = 0.39, *P* = 0.68) or adult (Log_10_-transformed; F_3,34_ = 1.09, *P* = 0.366) *H. bakeri* did not vary among treatment groups. While sex ratio did not differ from 1.0 for L_4_ (LCN: 1.06 ± 0.16, t = 0.36, df = 7, *P* = 0.73; HCN: 1.27 ± 0.37, t = 0.75, df = 7, *P* = 0.48; N: 0.99 ± 0.10 t = 0.113, df = 7, *P* = 0.91), sex ratio of adult worms was female biased for SI (t = 3.36, df = 7, *P* = 0.012) and N (t = 4.62, df = 14, *P* < 0.0001) treatment groups but not for CN (t = 0.09, df = 7, *P* = 0.93) or NC (t = 2.09, df = 6, *P* = 0.08) treatment groups (back-transformed mean ± upper/lower standard error, CN: 1.030 ± 0.001/0.001; NC: 0.762 ± 0.002/0.001; SI: 0.674 ± 0.002/0.002; N: 0.758± 0.003/0.003). 

**Figure 3:**
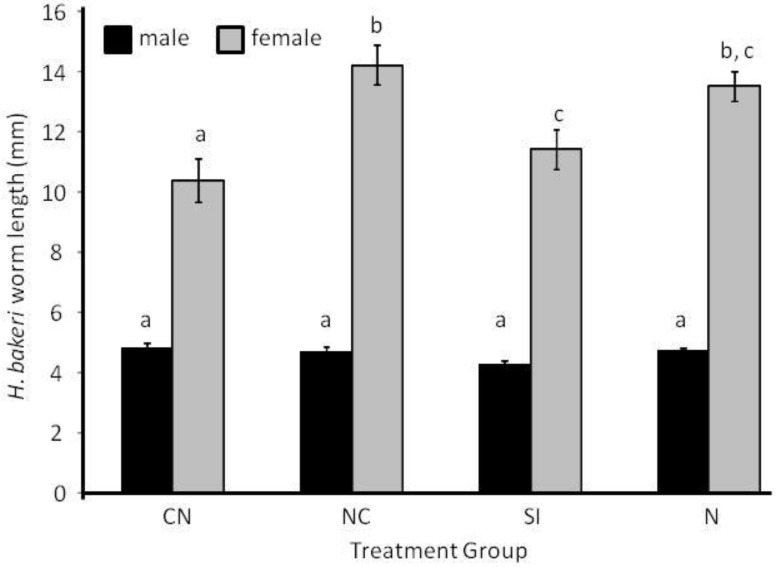
Length of male and female *Heligmosomoides bakeri* nematode worms taken from mice in different treatment groups in Experiment 2 (CN: cestode then nematode; NC: nematode then cestode; SI: simultaneous inoculation; N: nematode only). Within a sex, bars that share the same letter do not differ from each other at *P* < 0.05. Values are means ± 1 standard error.

Length of adult female *H. bakeri* worms was shortest when worms were from CN mice and longest when from N and NC mice (F_3, 34.2_ = 7.39, *P* = 0.001; Figure 3), but length of male *H. bakeri* worms was similar among all treatment groups (F_3,35.1_ = 2.33, *P* = 0.091; Figure 3). Average individual *H. microstoma* dry mass was greater for worms from LCN than HCN mice (F_1,14_ = 15.36, *P* = 0.002; Figure 2) because dry mass was negatively correlated with infection intensity (R^2^ = 0.78, y = −2.1x + 29.9; *P* < 0.0001). Despite the lack of difference in infection intensity of *H. microstoma* during Experiment 2, *H. microstoma* worms from C or CN mice weighed less than worms from SI mice (Log_10_ transformed; F_3,41_ = 5.89, *P* = 0.002; Figure 2) with worms from NC mice having intermediate mass.

**Figure 4:**
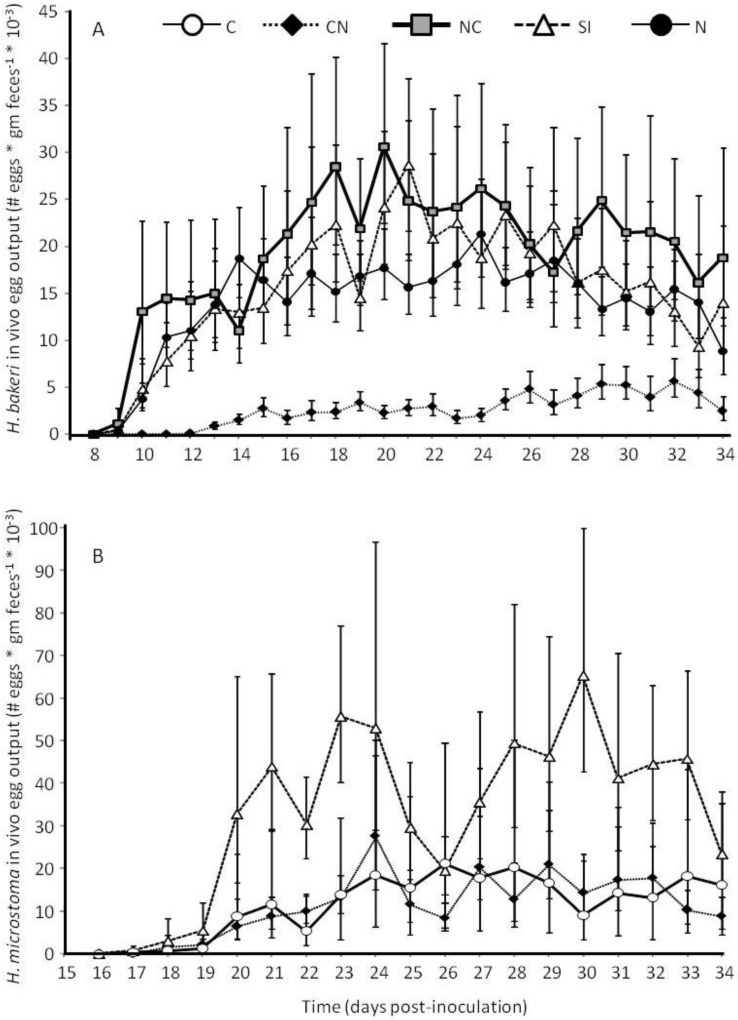
*In vivo* fecal egg output for the nematode *Heligmosomoides bakeri* (**A**) and the cestode *Hymenolepis microstoma* (**B**) from mice in different treatment groups in Experiment 2 (C: cestode only; CN: cestode then nematode; NC: nematode then cestode; SI: simultaneous inoculation; N: nematode only). Values are means (backtransformed from Log_10_ (egg output + 10)) ± 1 standard error.

**Figure 5:**
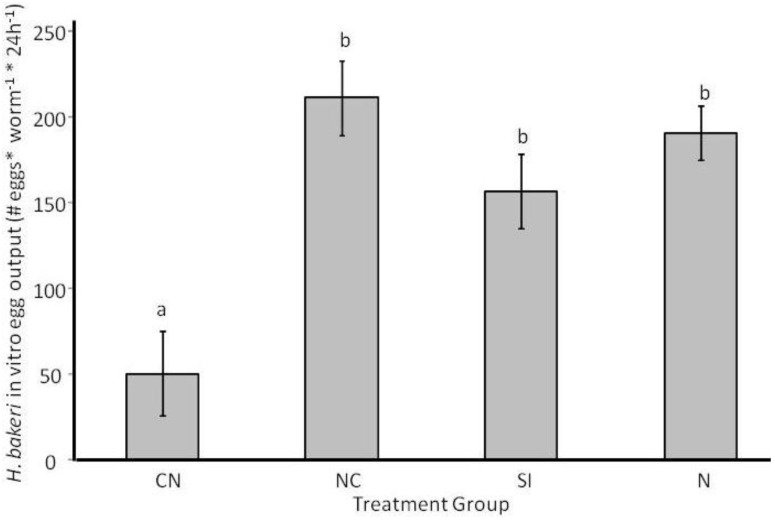
*In vitro* egg output of individual nematode *Heligmosomoides bakeri* worms taken from mice in different treatment groups in Experiment 2 (CN: cestode then nematode; NC: nematode then cestode; SI: simultaneous inoculation; N: nematode only). Bars that share the same letter do not differ from each other at *P* < 0.05. Values are estimated marginal means ± 1 standard error.

*H. bakeri* *via* fecal egg output varied over time (Log_10_ (egg output+10)–transformed; F_6.2,204.4_ = 14.26, *P* < 0.0001) with a significant interaction between time and treatment group (F_18.6,204.4_ = 5.31, *P* < 0.0001) because worms from CN mice did not show the increased egg output over time that occurred for worms of other treatment groups (Figure 4A). After accounting for *H. bakeri* infection intensity as a covariate (F_1,33_ = 18.9, *P* < 0.0001), CN mice had fewer *H. bakeri* eggs in their feces than mice in the N, SI or NC treatment groups which did not differ from each other (F_3,33_ = 12.65, *P* < 0.0001; Figure 4A). When individual *H. bakeri* worms were isolated to measure *in vitro* reproduction, we found that, for *H. bakeri* females that produced at least one egg, worms from CN mice produced the fewest eggs but worms from the other treatment groups did not differ from each other (F_3,34_ = 10.07, *P* < 0.0001; Figure 5). The same pattern of egg output was seen if all worms, including those that produced zero eggs, were considered (data not shown). *H. microstoma* *in vivo* fecal egg output varied with time (Log_10_ (egg output+10)–transformed; Greenhouse-Geisser F_7.4,216_ = 36.95, *P* < 0.0001) similarly for all treatment groups (Greenhouse-Geisser F_14.9,216_ = 0.77, *P* = 0.71; Figure 4B). Overall, *H. microstoma* from SI mice produced more eggs than *H. microstoma* from either C or CN mice which did not differ from each other (F_2,29_ = 6.27, *P* = 0.005; Figure 4B).

### 2.2. Host Morphology & Liver Function

Body mass of mice increased in both experiments (Experiment 1: F_1,21_ = 9.52, *P* = 0.006, Experiment 2: F_1,54_ = 349.22, *P* < 0.0001; Table 1), and the change in mass was similar across each treatment group (Experiment 1: F_2,21_ = 0.11, *P* = 0.897, Experiment 2: F_4,54_ = 2.12, *P* = 0.091). Overall, mouse body mass did not differ among treatment groups for Experiment 1 (F_2,21_ = 0.90, *P* = 0.421) or Experiment 2 (F_4,54_ = 0.98, *P* = 0.427; Table 1). 

Small intestine mass did not vary among treatment groups when mice were examined with L_4_ *H. bakeri* present (F_2,21_ = 0.31, *P* = 0.74; Table 1). However, during an adult *H. bakeri* infection, mouse small intestine dry mass was heaviest for CN and SI mice and lightest for single-infected mice (F_4,53_ = 17.21, *P* < 0.0001; after correcting for host body mass at dissection as a covariate, F_1,53_ = 110.35, *P* < 0.0001; Table 1). Despite these differences in small intestine mass, small intestine length did not differ among treatment groups (F_4,53_ = 2.09, *P* = 0.095) when corrected for host body mass at dissection as a covariate (F_1,53_ = 6.50, *P* = 0.014; Table 1). In Experiment 1, the number of *H. microstoma* scolices did not affect bile duct mass (R^2^ = 0.0004, slope = 0.0, *P* = 0.945) and bile duct mass also did not differ between LCN and HCN mice (F_1,14_ = 0.05, *P* = 0.826). Therefore, LCN and HCN data were pooled and compared against nematode-only infected mice (N) which showed that nematode only mice had 83% lighter bile duct mass compared to coinfected mice (F_1,21_ = 7.48, *P* = 0.012; Table 1). In Experiment 2, bile duct mass varied among treatment treatments (Log_10_ transformed; F_4,55_ = 74.21, *P* < 0.0001). Specifically, mice given only *H. microstoma* (C) or *H. microstoma* first (CN) had similar bile duct mass that was larger than the other two coinfected groups (NC or SI) which did not differ from each other and *H. bakeri* only infected mice (N) had the smallest bile ducts that were different from all other treatment groups (Table 1). The number of *H. microstoma* scolices did not affect bile duct mass for mice in Experiment 2 that had at least 1 scolex (R^2^ = 0.046, y = 0.001x + 0.014, *P* = 0.159). 

**Table 1:** Laboratory mouse (*Mus musculus*) morphological responses to *Heligmosomoides bakeri* and/or *Hymenolepis microstoma* infection for mice from treatment groups for Experiment 1 (LCN: low cestode infection, then nematode; HCN: high cestode infection, then nematode; N: nematode only) and Experiment 2 (C: cestode only; CN: cestode then nematode; NC: nematode then cestode; SI: simultaneous inoculation; N: nematode only). Values are means ± standard error. For small intestine dry mass for Experiment 2, means that share the same superscript letters are not significantly different from each other at *P* < 0.05.

	Initial body mass (g)	Final body mass (g)	Bile duct dry mass (g)^a^	Small intestine dry mass (g)^b^	Small intestine length (mm)^b^
**Experiment 1**					
LCN	30.1 ± 1.0	31.3 ± 1.0	0.023 ± 0.008	0.419 ± 0.019	N/A ^c^
HCN	28.7 ± 0.9	29.6 ± 1.1	0.025 ± 0.005	0.399 ± 0.027	N/A ^c^
N	28.8 ± 0.8	30.1 ± 0.6	0.007 ± 0.003	0.463 ± 0.097	N/A ^c^
**Experiment 2**					
C	29.7 ± 0.5	33.8 ± 0.4	0.026 ± 0.002	0.476^d^ ± 0.007	442 ± 8
CN	28.8 ± 0.4	33.9 ± 0.5	0.026 ± 0.004 (U)/ 0.003 (L)	0.556^f^ ± 0.011	396 ± 14
NC	28.7 ± 0.7	32.2 ± 0.9	0.012 ± 0.002	0.490^d,e^ ± 0.013	431 ± 16
SI	28.5 ± 0.8	31.9 ± 1.0	0.012 ± 0.002	0.528^e,f^ ± 0.011	422 ± 14
N	29.5 ± 1.5	33.4 ± 1.8	0.003 ± 0.000	0.458^d^ ± 0.008	426 ± 10

^a^ For Experiment 2, values are backtransformed from Log10; standard error was asymmetrical for CN (upper (U) and lower (L) values are shown); ^b^ For Experiment 2, values are estimated marginal means adjusted for a covariate of mouse body mass = 33.30 g; ^c^ Data not measured for Experiment 1.

**Figure 6:**
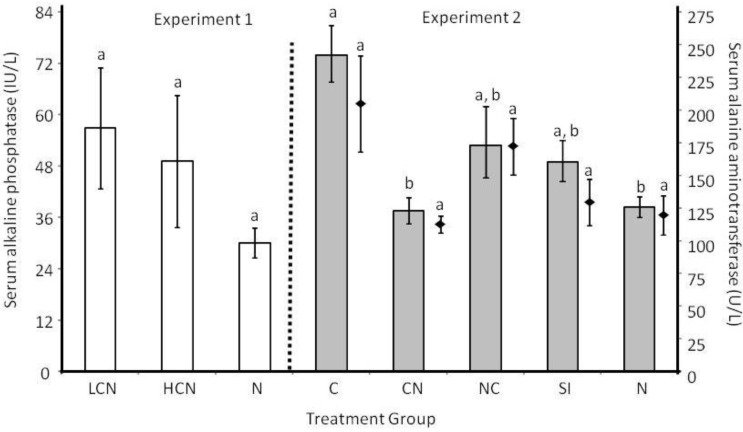
Serum alkaline phosphatase (ALP; IU/L) shown in bars and serum alanine aminotransferase (ALT; U/L) shown by diamonds for different treatment groups (LCN: low cestode infection, then nematode; HCN: high cestode infection, then nematode; N: nematode only (both experiments); C: cestode only; CN: cestode then nematode; NC: nematode then cestode; SI: simultaneous inoculation). Within an experiment, bars or diamonds that share the same letter do not differ from each other at *P* < 0.05. Values are means (backtransformed from Log_10_ for ALP in Experiment 2) ± 1 standard error.

Liver function did not vary among treatment groups as measured by ALP after 27d of *H. microstoma* infection in Experiment 1 (F_2,19_ = 0.58, *P* = 0.572; Figure 6). However, after a longer *H. microstoma* infection in Experiment 2, ALP levels varied among treatment groups (Log_10_ transformed; F_4,55_ = 10.59, *P* < 0.0001; Figure 6) because ALP for mice infected with *H. microstoma* only (C) was greater than for mice infected with *H. bakeri* only (N) or *H. microstoma* followed by *H. bakeri* (CN), with mice from other treatment groups (NC and SI) having intermediate values. ALP levels did not vary with number of *H. microstoma* scolices for mice with at least one scolex (R^2^ = 0.002, y = 0.63x + 59.2, *P* = 0.765). There was no difference in ALT production among treatment groups (F_4,55_ = 1.84, *P* = 0.135) or when only mice with at least one *H. microstoma* scolex were included (F_3,41_ = 1.37, *P* = 0.267; Figure 6). ALT levels were not related to the number of scolices (R^2^ = 0.008, y = −5.01x + 206.2, *P* = 0.569) for mice with at least one scolex. 

### 2.3. Discussion

When parasites share a host, there are numerous ways in which they may affect each other. Having another parasite species in the host can alter the parasite’s environment indirectly (e.g., *via* bile concentrations along the small intestine) or directly (e.g., occupation of the same host space where physical contact is possible). Data from our experiments show that order of coinfection influences life history traits of both *H. bakeri* and *H. microstoma* and that it is not always advantageous to be the first, or even the only, parasite in the host. 

#### 2.3.1. Larval Establishment & Growth to Adult Size.

The presence, but not the absolute number, of *H. microstoma* hindered larval establishment of *H. bakeri* and those that did survive were embedded more distally in the small intestine. One possible explanation is that the physical presence of *H. microstoma* strobila in the small intestine allowed bile to disperse further *via* capillary action. We did not measure individual *H. microstoma* worm length, but it would be interesting to determine the relative amount of bile in the proximal portion of the small intestine with and without *H. microstoma* worms. Alternatively, it has long been known that *Hymenolepis diminuta* infection decreases intestinal pH [40] and alters microflora composition [41] and the same may be true for *H. microstoma*. Low pH will increase secretin secretion [42] which, in turn, elicits increased production of bicarbonate-rich bile [43]. Therefore, bile may occur more distally in the small intestine because of greater total bile secretion, irrespective of *H. microstoma* acting as a physical corridor to enable bile dispersion. Although both parasites appear to use bile as a cue for site selection [32,44], during a simultaneous infection, larval *H. microstoma* likely would not affect bile flow and, therefore, are not predicted to affect *H. bakeri* larval establishment or site selection during a simultaneous infection. This relationship needs empirical examination which may help to elucidate why infection intensity of *H. bakeri* adults did not vary between the SI and NC groups. In addition to physiochemical changes, the host immune response elicted by *H. microstoma* may have damaged *H. bakeri* larvae which decreased establishment and also may have hindered subsequent adult survival. *Hymenolepis* sp. produce excretory/secretory (ES) products; however, to our knowledge, effects of *Hymenolepis* sp. ES products on nematode larvae are unstudied. ES from *Hymenolepis diminuta* decrease host immune responses [45,46]. If this also occurs for *H. microstoma*, then adult tapeworms should have made the intestinal environment more, not less, favorable for *H. bakeri*. 

In partial support of our hypothesis, growth of female, but not male, *H. bakeri* worms was influenced by *H. microstoma* whereby being alone (N), or establishing first (NC), in the small intestine was the best environment for *H. bakeri* females to grow. Adult site selection can correlate with final adult size, as females in more distal regions were typically shorter than females in the proximal region (data not shown). Results from simultaneous infections indicate that growth of female *H. bakeri* to adult size decreased in the presence of larval *H. microstoma* independent of site selection because site selection of adults in the SI and NC treatment groups did not differ yet females from the NC treatment group were 24% longer than females from the SI treatment group. Presence of coinfection also altered female but not male adult length during a *Heligmosomoides bakeri* and *Trypanosoma congolense* coinfection, but in this case females of coinfected mice were longer than females from mice infected only with *H. bakeri* [47]. Why coinfection alters female but not male growth is unclear, but it may indicate that males and females rely on different cues for growth. Two general determinants of final adult size are growth rate and duration of the growth period. Experiments to measure male and female lengths throughout the L_4_ and early adult stages during coinfection could resolve if males and females differ in one or both of these parameters. 

While we observed the classic crowding effect in *H. microstoma*, this was not the only determinant of adult worm mass. Although *H. microstoma* had poor growth in a high inflammatory host environment during coinfection with the expulsion phase of *Trichinella spiralis* [48], the relatively low inflammatory environment of a mature *H. bakeri* infection did not yield maximal *H. microstoma* mass. This is evidenced by the observation that, although there were similar infection intensities among treatment groups, *H. microstoma* mass was not greater when a mature *H. bakeri* infection occurred before *H. microstoma* were given (NC) when compared to mice infected only with *H. microstoma* (C) or when *H. microstoma* were given before *H. bakeri* (CN). Instead, *H. microstoma* grew heaviest when mice received both parasites simultaneously (SI). While the data do not support our hypothesis that immunosuppressive factors produced by adult *H. bakeri* benefit *H. microstoma* growth, it appears that either indirect host changes induced by *H. bakeri* L_4_ (immune or otherwise), or direct interactions between larval stages when both parasites are in the lumen or mucosal layers of the small intestine [49,50] produce the most favorable environment for *H. microstoma* growth. An evaluation of *H. microstoma* growth before and after *H. bakeri* larvae embed at the muscularis and/or *H. microstoma* move into the bile duct may resolve if direct larval parasite interactions influence final adult body mass of *H. microstoma*. Alternatively, when *H. bakeri* moves through the small intestine layers to enter the lumen as adults, there may be residual mechanical damage in the small intestine that is sensed by *H. microstoma* to then inhibit growth. It would be informative to determine if waiting longer after *H. bakeri* infection before inoculation with *H. microstoma* would result in better *H. microstoma* growth.

#### 2.3.2. Adult Survival and Reproduction

Although the number of L_4_ worms was clearly less when *H. microstoma* occurred first, there were only marginally fewer adult *H. bakeri* worms in mice in the CN treatment group compared to other treatment groups; therefore, subsequent differences in survival among treatment groups must have occurred during the late L_4_ to early adult stages. If ES products of adult *H. microstoma* worms had additional effects on adult *H. bakeri* worm survival then there also should have been differences between the NC and SI treatment groups compared to *H. bakeri*-only infected mice (N), which was not the case. Larval site selection had a long-term impact on adult site selection of *H. bakeri* where worms from CN mice were located more distally in the small intestine compared to other treatment groups. It is unclear why adult *H. bakeri* in CN mice would not simply migrate proximally in the small intestine. Perhaps presence of the adult *H. microstoma* worms physically interfered with *H. bakeri* worm movement or induced some other physiochemical changes in the small intestine that interrupted normal cues for site selection of adult *H. bakeri*. Experimental studies are needed to elucidate the mechanism of worm movement and site selection by adult *H. bakeri* in the present of mature *H. microstoma* worms. Although sex ratio was similar among treatment groups, sex ratio was female biased when mice received a simultaneous or nematode only infection. Adult females are more resistant than males to exogenous reactive oxygen species [51] which could explain the occurrence of female biased sex ratios previously observed in *H. bakeri*-only infections [51,52]. If coinfection alters host derived reactive oxygen species then this may be sufficient to cause deviation in the sex ratio. 

Except when *H. bakeri* larvae developed after a mature *H. microstoma* infection was present, the physical presence of *H. microstoma* did not impact egg deposition of adult female *H. bakeri*. This is evidenced by similar reproduction for worms from SI, NC and N mice and also because overall patterns of in vivo reproduction were maintained when female *H. bakeri* were isolated for in vitro measures where infection intensity is not a potentially confounding effect. Instead, our data provide indirect support for the negative relationship between female length and reproduction because average female length was shortest for worms from CN mice (although we did not measure length of individual worms that were used for reproduction measures). In an interspecific comparison of nematode species, female fecundity positively correlated with worm length [53]. If this trend holds within a single species, there should be strong selection for females to obtain their optimal size to maximize reproduction. If female length is a result of determinant growth that was altered in the presence of a mature *H. microstoma* infection, and if that length then determines reproduction, then *H. microstoma* will induce sustained negative consequences for lifetime reproductive success of *H. bakeri* females since *H. microstoma* are predicted to outlive *H. bakeri* if they have similar longevity as *H. diminuta* [54]. Further work to determine if adult length is an important predictor of female reproductive success for *H. bakeri* would elucidate the potential evolutionary importance of coinfection on this life history trait. We are unaware of any studies of the relationship between *H. bakeri* male length and reproduction (e.g., sperm count, sperm motility) or survival of individual worms. If *H. bakeri* male length does not impact life history, this may explain why male length was invariant among our treatment groups.

Contrary to our hypothesis, adult *H. microstoma* survival was impervious to presence of *H. bakeri*, regardless of the order of inoculation. We did not measure larval establishment of *H. microstoma* during simultaneous or mature *H. bakeri* infections, so it is unknown if final adult infection intensity is determined by similar larval establishment among treatment groups or by differential adult survival. 

Despite similar infection intensity, reproduction by and mass of *H. microstoma* was greatest when worms were in mice that received simultaneous infections (SI). Our data only partially support our hypothesis for greater *H. microstoma* reproduction during *H. bakeri* coinfection because *H. microstoma* reproduction was poor in CN mice even though *H. bakeri* were present. Therefore, greater egg production in SI mice was not solely a function of an immune-suppressed host environment caused by *H. bakeri*. That reproduction, but not survival, of *H. microstoma* was affected by coinfection illustrates the point that multiple life history traits should be measured to determine the importance of coinfection for particular parasite species.

#### 2.3.3. Host Morphological and Physiological Responses

Enlargement of bile duct tissue during *H. microstoma* infection, caused primarily by hyperplasia and increased fibrous tissue [35,55], is accompanied by liver lesions associated with leukocyte infiltration and degeneration of hepatocytes [56]. Host hepatobiliary changes are due, at least in part, from *H. microstoma* ES products because experimental induction of *H. microstoma* ES products induced lesion formation on mouse livers similar to those seen during actual *H. microstoma* infection [56]. If *H. bakeri* can decrease production of *H. microstoma* ES products, this may explain our observation that the increase in bile duct mass during *H. microstoma*-only infection was reduced by approximately 50% when *H. bakeri* were given first (NC) or simultaneously (SI). However, if *H. bakeri* occurred after a mature *H. microstoma* infection was present, then *H. bakeri* could not reverse the existing morphological changes to bile duct tissue. Although bile duct mass was similar for mice given *H. microstoma* first in both experiments, only mice in Experiment 2 showed increased alkaline phosphatase (ALP) levels. Infection duration was 27d in Experiment 1 but ranged up to 55d for mice in Experiment 2; therefore, the overall longer *H. microstoma* exposure permitted higher serum ALP levels. Worm mass was not important to bile duct occlusion since mice with *H. microstoma*-only infections (C) had the highest ALP levels but the lightest worms; likely this is because worm mass is primarily determined by strobila length and width and strobila are within the small intestine. Despite evidence of bile duct occlusion in CN mice, liver damage was not sufficient to increase levels of ALT. Longer infection durations with *H. microstoma* may result in more advanced liver pathology to cause detectable changes in serum ALT. Contrary to our hypothesis, *H. microstoma* worm number did not affect liver enzymes, perhaps because liver lesions can form in the presence of just one *H. microstoma* worm [35]. Measures of bile duct occlusion and liver function are important because changes in liver function may influence host metabolic state and thereby influence capacity for immune responses or other metabolically expensive activities critical for mouse survival and reproduction.

Changes in mouse body mass did not vary among any treatment groups for either experiment, and all mice showed the expected mass increase over time. Therefore, even if increased metabolic rate occurred during coinfection, as has been demonstrated for single infections of *H. microstoma* [57] and *H. bakeri* [58], mice were able to compensate (e.g., *via* decreased activity or increased food intake) to maintain body mass. While small intestine length was similar for single and coinfections, our hypothesis that coinfected mice would have greater masses than single-infected mice was supported. Effects of immune cell infiltration on tissue mass have not been directly examined, but our data are consistent with previous work showing increased mucosal/submucosal masses during *H. bakeri* infection which likely reflects changes in glucose transport and increased metabolic rate [58]. Such phenotypic plasticity of the small intestine in our coinfected mice demonstrates that increasing intensity of demands (e.g., energy to fuel immune responses to two parasites) can elicit ever increasing masses of the small intestine [59]. 

## 3. Experimental Methods

### 3.1. Experimental Design

For each experiment, there was one independent variable of “treatment group” and numerous dependent variables to assess parasite life history and host hepatobiliary and small intestine responses. The Institutional Animal Care and Use Committee (IACUC) of California State University San Marcos approved all procedures. 

#### 3.1.1. Experiment 1

**Figure 7:**
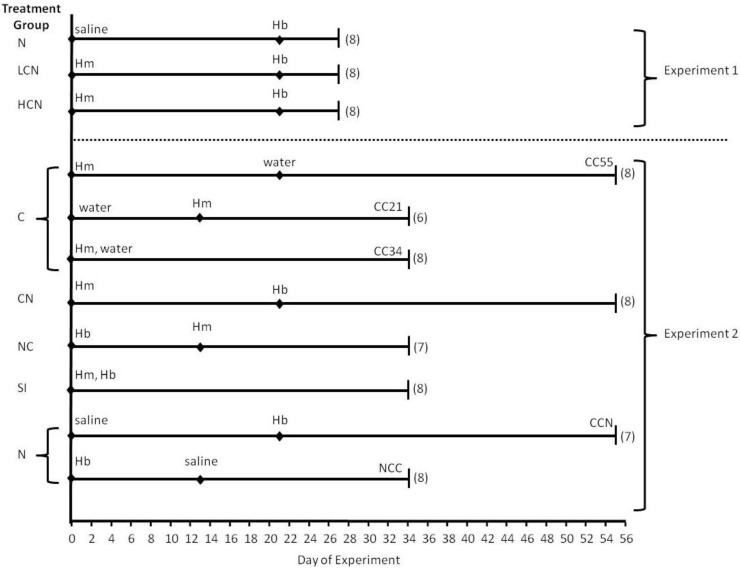
Timeline (days) of experimental procedures for *Hymenolepis microstoma* and *Heligmosomoides bakeri* coinfection experiments. Final treatment groups are denoted on the left for Experiment 1 (N: nematode only; LCN: low cestode infection, then nematode; HCN: high cestode infection, then nematode) and Experiment 2 (C: cestode only; CN: cestode then nematode; NC: nematode then cestode; SI: simultaneous inoculation; N: nematode only) with sample size (# mice) shown in parentheses. Abbreviations on each line are as follows: Hb = *Heligmosomoides bakeri*, Hm = *Hymenolepis microstoma*, CC55 = control cestode 55 day infection duration, CC34 = control cestode 34 day infection duration, CC21 = control cestode 21 day infection duration, CCN = control cestode then nematode, NCC = nematode then control cestode, saline = saline administered as a control for cestode inoculation, water = water administered as control for nematode inoculation.

Experiment 1 evaluated the effects of coinfection on fourth larval stage (L_4_) *H. bakeri* and adult *H. microstoma* whereby mice were dissected 6d post-*H. bakeri* infection and 27d post-*H. microstoma* infection (Figure 7). There were 3 treatment groups: (1) low intensity cestode infection (4 *H. microstoma* cysticercoids) followed by nematode infection (LCN), high intensity cestode infection (12 *H. microstoma* cysticercoids) followed by nematode infection (HCN), and (3) nematode only infection (N) where mice received 0.9% saline as a control for the cestode inoculation. For each treatment group, *H. bakeri* infection was given 21 days after *H. microstoma* or control inoculation and all mice received 300 infective stage larvae (L_3_).

#### 3.1.2. Experiment 2

Experiment 2 evaluated the effects of coinfection on adult *H. bakeri* and adult *H. microstoma* whereby all mice were dissected 34d post-*H. bakeri* infection with 300 L_3_ and either 21d, 34d or 55d post-*H. microstoma* infection with 12 cysticercoids (the same infection intensity as the HCN group from Experiment 1) (Figure 7). To verify that age of *H. bakeri* L_3_ would not affect life history measures, we tested two groups of control mice: the nematode followed by control cestode (NCC) group received *H. bakeri* that were six days older than L_3_ used to infect mice in the control cestode followed by nematode (CCN) group. Dependent variables did not differ for CCN and NCC; therefore, these were pooled into one nematode only treatment group (N) for all analyses presented herein. The coinfected groups in Experiment 2 included a nematode followed by cestode infection (NC), a cestode followed by nematode infection (CN) and a group that received both parasites simultaneously (SI). We tested if duration of *H. microstoma* infection influenced dependent variables using three control cestode groups: cestode only for 21d (CC21) which was a control for the NC coinfected group, cestode only for 34d (CC34) which was a control for the SI coinfected group, and cestode only for 55d (CC55) which was a control for the CN coinfected group. Because dependent variables did not differ among CC21, CC34 and CC55, they were pooled into one cestode only group (C) for all analyses presented herein. Therefore, Experiment 2 had five final treatment groups. 

### 3.2. Host Maintenance and Parasite Inoculations

Male Swiss Webster mice (Harlan, Indianapolis, IN, USA) 5.5–7 weeks old were housed in groups of three to four, maintained on a 14:10 light:dark cycle, and provided water and food (Purina Mouse Chow #5001) *ad libitum*. Our original stock of *H. microstoma* was graciously supplied by Dr. Jerzy Behnke at the University of Nottingham and was maintained in our lab by passage through confused flour beetles (*Tribolium confusum*) and Swiss Webster laboratory mice. Using a dissecting microscope, we dissected *H. microstoma* cysticercoids from *T. confusum* that had been placed in 0.9% saline. To inoculate mice, cysticercoids, along with 20–25 µL saline, were taken into a 0–200 µL pipette tip and then dispensed into the mouse’s mouth. Following each inoculation, the pipette tip was flushed with saline, contents were inspected using a dissecting microscope, and any remaining cysticercoids were re-administered as described above. For both experiments, flour beetles were infected with *H. microstoma* eggs between 39-80 days prior to cysticercoid dissection. *H. bakeri* was cultured using standard methods [58] and maintained in non-experimental Swiss Webster mice. Within each experiment, all L_3_ were from a single culture where L_3_ age was less than three months. To inoculate experimental mice with *H. bakeri*, L_3_ (suspended in tap water) were dispensed into the mouse’s mouth *via* a 0–200 µL pipette; nematode control mice received an equal volume of tap water. 

### 3.3. Parasite Life History and Host Tissue Response Variables

Mouse body mass was measured just prior to infection and on day of dissection. For Experiment 2, *in vivo* parasite egg output was counted from 1–3 fecal pellets that were collected daily from each mouse (starting at 8d post- *H. bakeri* inoculation or 12d post- *H. microstoma* inoculation). Number of eggs was counted using floatation with saturated sucrose and egg output per gram of feces was calculated separately for each parasite by the following equation: ([volume of water + volume of sucrose]/feces mass) * (# eggs per grid/volume of grid). For each mouse sample, two grids of a McMaster slide were counted and the average was used as the eggs per gram of feces for each parasite. Just prior to euthanasia, 200 µL of blood was collected from the ocular sinus of anesthetized (*via* inhaled isoflurane) mice using 70 µL heparinized capillary tubes. Blood was centrifuged for 15 minutes at 9,500 × g and resulting plasma was aliquoted and frozen at −80 °C until used for ALT or ALP kinetic colorimetric assays. ALT (units per liter, U/L) was measured on a microplate reader at 340nm for a 15 µL plasma sample after 5 minutes of reaction time according to manufacturer instructions (ALT reagent C164-01, calibrator C1200-10, Catachem Inc., Bridgeport¸ CT, USA; assay was verified using manufacturer controls of Catatrol I C1200-11 and Catatrol II C1200-12, Catachem Inc., Bridgeport¸ CT, USA). ALP (international units per liter, IU/L) was measured on a microplate reader at 405 nm for a 10 µL plasma sample after 4 minutes of reaction time according to manufacturer instructions (DALP-250, BioAssay Systems, Hayward, CA, USA). 

After mice were euthanized, the small intestine and the combined cystic duct and common bile duct (hereafter called “bile duct”) were removed. The bile duct was opened longitudinally and inspected using a dissection microscope and all *H.* *microstoma* scolices and worm tissue were removed to a separate Petri dish. The small intestine was cut into 6 equal-length segments where segment #1 began immediately after the stomach at the pyloric sphincter and segment #6 ended immediately proximal to the cecum at the ileocecal valve. Each segment was measured to the nearest 1mm (for Experiment 2), placed in 0.9% saline, and opened longitudinally. Total small intestine length was calculated as the sum of the six segments. Pieces of *H. microstoma* were removed, added to *H. microstoma* tissue taken from the bile duct, and all scolices were counted. Adult and L_4_ *H. bakeri* were removed from each segment using watchmaker forceps (L_4_ were removed from the serosal side and adults from the luminal side) under a dissecting microscope. All *H. bakeri* worms were counted and sexed. *H. bakeri* sex ratio was calculated as the number of males divided by the number of females. In Experiment 2, length of a sub-sample of *H. bakeri* worms (10 male, 10 female) from each mouse was measured to the nearest 0.01 mm using an ocular micrometer fitted to a dissecting microscope. Another set of 12 adult *H. bakeri* female worms per mouse was used to measure *in vitro* egg output. Each worm was individually placed in a well of a 48-well plate that contained 500 µL of RPMI media that was supplemented with 1X penicillin/streptomycin and 0.08% amphotericin B. The plates were incubated in 5% CO2:95% N for 24 h at 37 °C. After 24 h, female worms were removed, well contents were mixed by pipetting and eggs were counted in a 350µL sample from each well using flotation with an equal volume of saturated sucrose. Egg output was calculated by the following equation: # eggs per grid/volume of grid * 2 (a dilution factor to account for volume of saturated sucrose) * volume of media. One grid of a McMaster slide was counted per worm. The bile duct, *H. microstoma* worms, and each intestinal segment were dried at 58 °C for 48 h to a constant mass and were then weighed to the nearest 0.0001 g to determine dry mass. Average individual *H. microstoma* worm dry mass was calculated as the dry mass of all worms combined divided by the number of scolices per mouse. Total small intestine dry mass was calculated as the sum of dry masses of the six segments.

### 3.4. Statistical Methods

We tested each variable for homogeneity of variance and transformed data as necessary. When appropriate, covariates were examined and only retained in the statistical model if significant. Repeated measures ANOVA was used to test if host body mass at infection and at dissection varied among treatment groups, and for *in vivo* fecal egg output over time. For *Hymenolepis microstoma* worms, host *in vivo* fecal egg output was only evaluated starting at day 16 post-infection for C, CN and SI mice for 34 d of infection to allow for a repeated measures model without missing values. Total infection intensity for each parasite, sex ratio of *H. bakeri*, ALT, ALP, bile duct mass, small intestine mass, small intestine length, and average individual *H. microstoma* mass were analyzed by ANOVA. A one-sample T test was used to determine if sex ratio differed from 1.0 (*i.e.*, an equal number of male and female worms). Infection intensity of *H. microstoma* in the high versus low infection treatments in Experiment 1 was tested with an adjusted Student’s T test without assumption of equal variances (because transformations would not yield homogeneity of variances). The distribution of *H. bakeri* worms in the six small intestine segments was analyzed with a one-factor multivariate analysis of variance (MANOVA) with segment number as the within subjects variable and the number of worms as the between subjects variable. Length of *H. bakeri* worms and *in vitro* egg output were analyzed using nested ANOVA (nested by mouse ID) because individual *H. bakeri* worms from each mouse could not be considered independent. Linear regression was used to determine if bile duct mass, ALP and ALT levels were affected by the number of *H. microstoma* scolices. When appropriate, post-hoc analyses used Tukey’s least significant difference or a comparison of 95% confidence intervals (unless otherwise stated). All data were analyzed with SPSS/PASW and considered significant at *P* < 0.05.

## 4. Conclusions

The negative impacts of a mature *H. microstoma* infection on *H. bakeri* included low L_4_ survival, poor growth leading to shorter adult female worms, and reduced reproduction; these negative impacts were largely nonexistent, however, if *H. microstoma* was introduced after or at the same time as *H. bakeri*. Conversely, *H. microstoma* generally benefitted when entering a host simultaneously with *H. bakeri* infection as evidenced by better growth to become heavier adults and greater reproduction compared to being in a host first or alone. Experimental designs that include various orders of infection can allow researchers to predict and test potential mechanisms that may influence the role of coinfection on different stages of the parasites’ life cycles. While our data cannot distinguish between the roles of excretory/secretory products or bile in parasite survival and site selection, future experimental manipulations or in vitro tests may allow us to distinguish between the relative importance of these potential mechanisms on life history of *H. bakeri* and *H. microstoma*. Tests to examine host metabolism during coinfection would reveal if small intestine mass increases during coinfection are to maintain energy balance in the face of metabolically costly immune responses or are a function of other, possibly more direct, effects of these intestinal helminths on the host small intestine. Whether our observed differences are unique to the two parasite species used or are typical of a more general coinfection response by intestinal Cestodes and Nematodes can be resolved with further experimentation of additional parasite species within these two orders. 
